# Out of the shadows: women in global health leadership

**DOI:** 10.1017/gheg.2018.15

**Published:** 2018-10-03

**Authors:** Pascale Allotey

**Affiliations:** Director, United Nations University – International Institute for Global Health (UNU-IIGH)

In a call launched on International Women's Day in 2016, *Global Health, Epidemiology and Genomics* (*GHEG*) was one of the first peer-reviewed journals to invite submissions that specifically explored the state of and reasons behind the gender imbalance in science and global health leadership [1]. The submissions highlighted the competing responsibilities inherent in gender roles that hindered leadership opportunities for women [2] as well as the extent of injustice, including violence and discrimination that deterred or actively prevented women from seeking or reaching higher levels of seniority [3, 4]. The papers noted the exceptional contributions that women have made in the field [2] and also reviewed a range of best practice examples of how change to achieve gender equality could be catalysed and sustained [4–6].

The intention of the *GHEG* call, and others like it, was to shed light on the pervasiveness of the gender power dynamics in all aspects of society, including in the scientific community. There is strong evidence to demonstrate that the exclusion of women as research participants, particularly in clinical research, has restricted our understanding of effective care [7, 8]. Furthermore, the lack of sex disaggregation and gender analysis in findings increases the risk of exacerbating inequality [9, 10]. The European Association of Science Editors has recently introduced the Sex and Gender Equity in Research (SAGER) guidelines as a framework to encourage a reversal of this gender blindness. A number of scientific journals and research funding agencies have responded by mandating reporting against the SAGER guidelines for both authors and reviewers of research submissions [11–13].

With a focus on women as current or potential producers of global health knowledge, the *GHEG* submissions raise two distinct but related issues. The first outlines the more general challenges faced by women in entering and maintaining careers in science and global health; the restrictions in educational opportunities for girls, the expectation of career breaks or drop-out in order to prioritise family and care giving roles and the structural and institutional factors that remain unforgiving of these career breaks and flexible work conditions [2]. While these challenges occur across disciplines, science and medicine have particular traditions of male dominance [14]. Evidence of this was epitomised by public comments from Nobel Laureat Tim Hunt about the distraction of having women in laboratories [14]. The lack of women on the research teams, unsurprisingly also translates to lower representation in authorship. There are gendered differences in opportunities to publish, women's representations in the editorial process and the quality of and reactions to the peer-review process [8, 15]. National Initiatives like the Athena SWAN Charter in the UK and the Science in Australia Gender Equity project (SAGE) and industry-specific ones like the Sex in Science (SiS) programme are responding to these challenges and have been well evaluated [6]. It would be refreshing to see journal editors extend the SAGER analyses to include authorship.

The second major issue relates specifically to *leadership*. While all the issues raised above are an important contributor to problems in the pipeline for career development, addressing leadership raises more challenges. Leadership covers a range of styles and responsibilities and could involve both formal recognition of seniority and expertise, as well as less formal positions that entail guiding and mentoring individuals or teams. Strategies for leadership development for women have focused largely on programmes that enable mentoring and personal coaching [2, 5, 6]. By definition, however, leadership roles are limited. This necessarily means that to increase the numbers of women in leadership positions, one would need to reduce the numbers of men; and this does pose a real threat to the hegemony. Recent debates in social media, for instance, note the concerns of some current male global leaders highlighting a perceived devaluation of their expertise in the efforts to identify women for high-level meeting panels. It is critical to note therefore that the pro-active promotion of women in leadership involves more than increasing the numbers of women to achieve parity, but also that it would need to involve a broader cultural change that involves working with both men and women to support an evolution in the nature of leadership, and in the power dynamics in gender relationships.

The increase in the calls for papers exploring women's experience of science and global health and in gender and health more broadly is an important step in supporting this evolution. As a strategy, the papers, which are largely observational studies and critical analyses, situate the evidence in contexts familiar to the target audiences of scientific journals. The more strident examples of whistleblowing [16] represent a ‘primitive, distressed cry for help’ [17]; and while divisive, also have the potential to accelerate cultural change. Noting the problem is the first step to addressing it. To that end, the call for papers in gender and global health will remain an open one for *GHEG*.
